# Decomposition and comparative analysis of health inequities between the male and female older adults in China: a national cross-sectional study

**DOI:** 10.1186/s12889-023-15814-5

**Published:** 2023-10-19

**Authors:** Zhe Zhao, Boyang Yu, Fangyuan Hu, Chao Zheng, Jing Gui, Jiahao Liu, Jinhai Sun, Jinhao Shi, Lei Yuan

**Affiliations:** 1 Department of Health Management, Second Military Medical University, Shanghai, China; 2 Department of Military Health Service, Second Military Medical University, Shanghai, China; 3 Department of Medical Service, Naval Hospital of Eastern Theater, Zhoushan, China; 4 Department of Acupuncture and Rehabilitation, the Second Affiliated Hospital of Guizhou, University of Traditional Chinese Medicine, Guiyang, China; 5 Department of Military Health Service Training, Second Military Medical University, Shanghai, China; 6 Xiamen Special Service Health Center of The Army, Xiamen, China; 7 Department of Research and Academic Management, Second Military Medical University, Shanghai, China

**Keywords:** Older adults, Self-rated health, Fairlie decomposition analysis, Health inequities

## Abstract

**Background:**

This study aimed to examine the factors influencing self-rated health (SRH) among Chinese older adults by gender differences and provide suggestions and theoretical references to help make policies for older adults’ health concerns by government agencies.

**Methods:**

Chinese Longitudinal Health Longevity Survey (CLHLS) in 2018 was adopted, the chi-squared test and the logistic regression analysis were performed to analyse self-rated health reported by Chinese female and male older adults and its influencing factors. In addition, Fairlie decomposition analysis was performed to quantify the contribution level of different influencing factors.

**Results:**

Among older adults, males (48.0%) reported a significantly higher level of good self-rated health than females (42.3%). Residence, body mass index (BMI), self-reported income, smoking, drinking, exercise, and social activity were the factors that influenced SRH reported by male and female respondents, with age, marital status and education reaching the significance level only in women. The Fairlie decomposition model can explain the underlying reasons for 86.7% of the gender differences in SRH, with self-reported income (15.3%), smoking (32.7%), drinking (42.5%), exercise (17.4%), social activity (15.1%) and education (-14.6%) being the major factors affecting gender differences in SRH.

**Conclusions:**

The study results can help promote the implementation of the Healthy China Initiative, inform intervention measures, and offer new proposals on creating policies for older adults’ health issues by the Chinese government to improve health equity.

**Supplementary Information:**

The online version contains supplementary material available at 10.1186/s12889-023-15814-5.

## Background

The global population is entering the ageing phase of the demographic cycle. Data from World Population Prospects 2019 released by the Population Division of the United Nations Department of Economic and Social Affairs showed that for the first time in history, persons aged 65 years or above worldwide outnumbered children under age five in 2018. By 2050, one in six people in the world will be over age 65 (16%), contrary to 1 in 11 in 2019 (9%). The number of persons aged 80 years or above is expected to increase threefold, from 143 million in 2019 to 426 million in 2050 [1]. China’s population ageing is also exacerbating. According to 2020 data of the seventh national census released by the Office of the State Council’s Leading Group in 2021, there were 19,06,35,280 people (13.50%) aged 65 or older in China, an increase of 4.63% compared to the 2010 sixth national census. The number of older adults aged 65 and above exceeded 7% in 30 provinces, except Tibet. Among these 30 provinces, the number of older adults aged 65 and above exceeded 14% in 12 provinces [2]. As the number of older adults continues to grow, there will be an increasing demand for healthcare among older adults, leading to concerns, such as the prolonged increase in medical expenses and burden China’s public financial expenditure. The Chinese government has implemented the Healthy China Initiative to promote people’s health. With a focus on disease prevention and health promotion, the initiative included 15 special campaigns to “intervene in health influencing factors, protect full-life-cycle health and prevent and control major diseases.” Among the 15 campaigns, the Older Adult Health Promotion Campaign is all about addressing the health issues of older adults. A few researchers have conducted studies on sex differences in SRH. Some studies indicated that older male adults reported better SRH than older female adults [3–5]. On the other hand, two studies demonstrated better perception of SRH among women [6, 7]. What’s more, two other studies suggested that there was no difference in SRH between male and female older adults [8, 9]. Therefore, further studies on factors influencing the health conditions of China’s older adults, especially those impacting gender differences among older adults’ health, will be instrumental in promoting health equity for older adults, delivering Chinese health authorities with better access to the healthcare demands of older adults, optimizing the allocation of healthcare resources, and improving the overall health for older adults.

To evaluate the health status of Chinese older adults, we adopted the self-rated health (SRH) indicator in this study. As a reliable indicator of overall physical fitness, SRH requires respondents to rate their health status on a scale from ‘very poor’ to ‘very good’, with the five-point scale being the most typical [3]. SRH is used to measure populations’ health status and inequality [10, 11] and predict the morbidity, mortality, and health service utilization rates [12–14]. Previous studies suggest that the determinants of self-rated health include age [15], activities of daily living (ADL) [16], self-compassion [17], socioeconomic status (SES), neighbourhood safety, and physical activity [18] among others. Further studies are required to assess the impact of gender differences on SRH. Takahashi and Baćak [19, 20] found gender differences in SRH in their study, whereas Campos and Rohlfsen [21, 22] did not identify an association between SRH and gender. The differences in study findings might be due to different study samples and analytical methods. Although the abovementioned studies explored the factors impacting population health, there are limited quantitative studies on the degree of contribution of each influencing factor. Fairlie decomposition analysis (FDA) is often applied in studies of the contribution of influences where the dependent variable is a dichotomous variable. Relevant studies suggested that [23, 24] Fairlie decomposition analysis can better quantify the contribution and significance level of various influencing factors. Therefore, FDA was performed to examine the factors impacting gender differences among older adults’ health and quantify the contribution level of each influencing factor. This study aims to help create policies for older adults’ health concerns by relevant departments and thus promote the implementation of the Healthy China Initiative.

## Methods

### Data sources

**Fig. 1:**
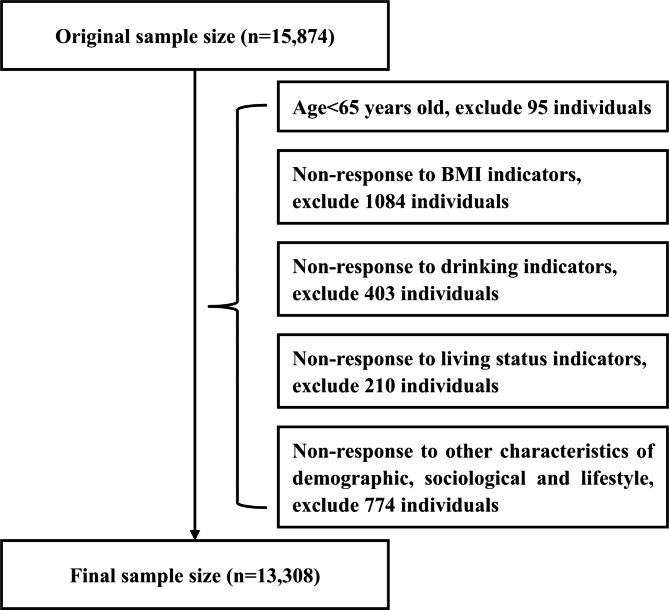
Flowchart of study participant

This study used data from the Chinese Longitudinal Health Longevity Survey (CLHLS) in 2018, published by the Center for Healthy Ageing and Development Studies at Peking University in 2020 [25]. The CLHLS is a nationwide survey conducted in a randomly-selected half of the counties and cities in 22 of the 31 provinces, covering about 85% of the total population of China. It included a total of 113,000 people in 23 provinces, municipalities, and autonomous regions (older adults of advanced age accounting for 67.40% of the total sample) [26, 27]. CLHLS in 2018 included a total of 15,874 people. According to the purpose of this study, we formulated the inclusion criteria for the study subjects: age ≥ 65 years old; demographic, sociological and lifestyle data such as age, gender, marital status, BMI, self-reported income, live status, and so on; SRH data. Based on these inclusion criteria, 13,308 respondents’ data were included in the study, with male respondents accounting for 45.27%, female respondents accounting for 54.73%, and older adults of advanced age (80 years or above) accounting for 63.73%. The data processing flow is shown in Fig. 1.

### SRH measurement

In CLHLS, SRH was used as a highly valid and reliable measure of health status among older adults [28–31]. The specific question included: ‘How do you rate your health at present?’ The answers included ‘very good’, ‘good’, ‘so so’, ‘bad’ and ‘very bad’. Referring to the numerical values assigned to the different categories of SRH status in previous relevant studies [31–34], this study assigned the value of 1 to ratings of ‘very good’ and ‘good’, and assigned the value of 0 to ratings of ‘so so’, ‘bad’ and ‘very bad’ (1 = Good SRH, 0 = Bad SRH). A sizable number of older adults were unable to answer questions due to a variety of difficulties, which also meant they were in poor health and quality of life. So, we assigned the value of 0 to rating of ‘not able to answer’.

### Variable description

The indicators affecting resident health status included demographic characteristics, social support, lifestyle, and socioeconomic status. Based on other studies on factors influencing health [9, 16, 19, 20], this study selected variables that might impact SRH in older adults from the CLHLS datasets. Social activity was assessed by activity participation, covering six items: tai chi, square dancing, visiting and interacting with friends, other outdoor activities, playing cards and/or mahjong, and social activities (organized). Each question was answered with five options: almost every day, not every day but at least once a week, not every week but at least once a month, not every month but sometimes, and never. Those who answered never in all activities were considered as not socializing and the results were classified as yes and no. The specific variable definitions and assignment of values are shown in an additional file [see Additional file 1].

### Statistical analysis

The SPSS software for Windows 21.0 was used for statistical analysis. General data were analysed using descriptive statistical methods; the chi-square test was performed to analyse the differences in SRH and gender differences in variable distribution among older adults at a significance level of α = 0.05. Logistic regression analysis was performed to analyse the SRH variables among Chinese older male and female adult.

#### Fairlie decomposition analysis (FDA)

The FDA was performed using the StataMP 16.0 statistical software to identify the gender differences in SRH among Chinese older male and female adults and its underlying cause. The decomposition technique was adopted to identify and quantify inter-group differences. The decomposition analysis identifies the contribution of independent variables to explain the differences across groups by calculating the average predicted probability change resulting from replacing one independent variable at a time for one group while other variables remain constant for the other group [35]. Relevant studies suggested that [34, 36–39] FDA for non-linear regression models can better quantify the contribution and significance level of different variables. The Fairlie decomposition model divides the study results into two segments: explained and unexplained [34, 40]. The explained part of the differences was caused by the observed variables in the study, whereas the unexplained part of the differences was caused by the differences in measured grouping variables and unmeasured variables. According to Fairlie [39], the decomposition of the nonlinear equation $Y=F\left(X\widehat{\beta }\right)$ can be written as:

1 $$\eqalign{{\stackrel{-}{Y}}^{a}-{\stackrel{-}{Y}}^{b}= & \left[\sum _{i=1}^{{N}^{a}}\frac{F\left({X}_{i}^{a}{\beta }^{b}\right)}{{N}^{a}}-\sum _{i=1}^{{N}^{b}}\frac{F\left({X}_{i}^{b}{\beta }^{b}\right)}{{N}^{b}}\right] \cr & + \left[\sum _{i=1}^{{N}^{a}}\frac{F\left({X}_{i}^{a}{\beta }^{a}\right)}{{N}^{a}}-\sum _{i=1}^{{N}^{a}}\frac{F\left({X}_{i}^{a}{\beta }^{b}\right)}{{N}^{a}}\right]} $$

$${\stackrel{-}{Y}}^{a} $$ and $${\stackrel{-}{Y}}^{b} $$ were the mean probabilities of the binary outcomes of depressive symptoms in the two groups, F was the cumulative distribution function of the logistic distribution, $${\stackrel{-}{Y}}^{a}-{\stackrel{-}{Y}}^{b} $$ represented the total variation due to group differences, $${N}^{a} $$ and $${N}^{b} $$ were the sample sizes of the two populations. The first term in parentheses in Eq. (1) represented the portion of the gap due to group differences in observed characteristics and the portion attributable to differences in estimated coefficients. The second term represented the portion due to differences in Y levels.

## Results

### General data of the respondents

Table 1 shows the results of descriptive statistical analysis comparing Chinese older male and female adults. We found that 55.09% of older adults were in ‘bad’ SRH, and 44.91% were in ‘good’ SRH. Women were more likely to report bad SRH outcomes, while those reported by men were more evenly distributed.

**Table 1:** Distribution of the variables in female and male respondents.

Variable	Female [*n* (%)]	Male [*n* (%)]	${\chi }^{2}$	*P*
SRH			42.1612	< 0.001
Bad	4198(57.64)	3134(52.02)		
Good	3085(42.36)	2891(47.98)		
Residence			12.9040	0.002
City	1535(21.08)	1405(23.32)		
Town	2388(32.79)	2001(33.21)		
Rural	3360(46.13)	2619(43.47)		
Age (years)			387.5999	< 0.001
< 70	702(9.64)	708(11.75)		
70–79	1666(22.88)	1751(29.06)		
80–89	1819(24.98)	1689(28.03)		
90–99	1544(21.20)	1329(22.06)		
≥ 100	1552(21.31)	548(9.10)		
Marital status			1376.1995	< 0.001
Married and living with spouse	2069(28.41)	3456(57.36)		
Widowed	5100(70.03)	2292(38.04)		
Other	114(1.57)	277(4.60)		
Living status			56.5756	< 0.001
Living with family	5701(78.28)	5025(83.40)		
Alone	1353(18.58)	839(13.93)		
Nursing home	229(3.14)	161(2.67)		
BMI (kg/m^2^)			126.7878	< 0.001
< 18.5	1435(19.70)	784(13.01)		
18.5–23.9	3614(49.62)	3221(53.46)		
24.0–27.9	1553(21.32)	1529(25.38)		
≥ 28.0	681(9.35)	491(8.15)		
Self-reported income			56.9181	< 0.001
Poor	773(10.61)	616(10.22)		
So so	5260(72.22)	4062(67.42)		
Rich	1250(17.16)	1347(22.36)		
Smoking			3797.7262	< 0.001
Current	306(4.20)	1760(29.21)		
Ever	287(3.94)	1708(28.35)		
Never	6690(91.86)	2557(42.44)		
Drinking			2093.3853	< 0.001
Current	435(5.97)	1566(25.99)		
Ever	349(4.79)	1207(20.03)		
Never	6499(89.24)	3252(53.98)		
Exercise			180.6712	< 0.001
No	5307(72.87)	3732(61.94)		
Yes	1976(27.13)	2293(38.06)		
Social activity			168.0327	< 0.001
No	2600(35.70)	1522(25.26)		
Yes	4683(64.30)	4503(74.74)		
Education (years)			1716.7933	< 0.001
0	4215(57.87)	1343(22.29)		
0–6	1494(20.51)	2256(37.44)		
≥ 7	1574(21.61)	2426(40.27)		

Women under the age of 90 years accounted for approximately 57% of all the included female respondents, whereas men under the age of 90 accounted for approximately 69% of all the male respondents. The proportion of women in older adults aged over 100 years was higher than that of men; 41.52% of the included respondents were married and living with their spouses, and 55.55% were widowed, with a higher proportion of widowed women in the study. Respondents who lived with family accounted for 80.60%.

The chi-squared test was performed to analyse the differences in categorical variables. The results showed statistically significant gender differences in the 12 variables included in this study (p < 0.05). Compared with women, men were characterized by better SRH, higher rates of married and living with spouse, higher likelihood of living with family, richer self-reported income, higher probability of smoking or drinking, and more exercise, social activity or years of education.

### Distribution of SRH variables in female and male respondents

Table 2 shows the distribution of SRH variables in female and male respondents. The results suggested statistically significant gender differences in the distribution of specific variables. There existed statistically significant SRH differences in the distribution of residence in female respondents; however, the differences were not statistically significant in male respondents. And the SRH differences in the distribution of marital status were statistically significant only in male respondents.

**Table 2:** Distribution of SRH variables under different statuses of SRH.

Variable	Female			Male		
	Bad SRH [*n* (%)]	Good SRH [*n* (%)]	*P*	Bad SRH [*n* (%)]	Good SRH [*n* (%)]	*P*
Residence			< 0.001			0.748
City	805(19.18)	730(23.66)		721(23.01)	684(23.66)	
Town	1437(34.23)	951(30.83)		1053(33.60)	948(32.79)	
Rural	1956(46.59)	1404(45.51)		1360(43.40)	1259(43.55)	
Age (years)			0.001			0.003
< 70	358(8.53)	344(11.15)		337(10.75)	371(12.83)	
70–79	945(22.51)	721(23.37)		874(27.89)	877(30.34)	
80–89	1072(25.54)	747(24.21)		892(28.46)	797(27.57)	
90–99	891(21.22)	653(21.17)		722(23.04)	607(21.00)	
≥ 100	932(22.20)	620(20.10)		309(9.86)	239(8.27)	
Marital status			0.535			0.015
Married and living with spouse	1182(28.16)	887(28.75)		1765(56.32)	1691(58.49)	
Widowed	2945(70.15)	2155(69.85)		1203(38.39)	1089(37.67)	
Other	71(1.69)	43(1.39)		166(5.30)	111(3.84)	
Living status			0.506			0.871
Living with family	3266(77.80)	2435(78.93)		2610(83.28)	2415(83.54)	
Alone	796(18.96)	557(18.06)		437(13.94)	402(13.91)	
Nursing home	136(3.24)	93(3.01)		87(2.78)	74(2.56)	
BMI (kg/m^2^)			< 0.001			< 0.001
< 18.5	912(21.72)	523(16.95)		473(15.09)	311(10.76)	
18.5–23.9	2060(49.07)	1554(50.37)		1680(53.61)	1541(53.30)	
24.0-27.9	854(20.34)	699(22.66)		734(23.42)	795(27.50)	
≥ 28.0	372(8.86)	309(10.02)		247(7.88)	244(8.44)	
Self-reported income			< 0.001			< 0.001
Poor	599(14.27)	174(5.64)		472(15.06)	144(4.98)	
So so	3094(73.70)	2166(70.21)		2183(69.66)	1879(64.99)	
Rich	505(12.03)	745(24.15)		479(15.28)	868(30.02)	
Smoking			0.005			< 0.001
Current	149(3.55)	157(5.09)		856(27.31)	904(31.27)	
Ever	163(3.88)	124(4.02)		951(30.34)	757(26.18)	
Never	3886(92.57)	2804(90.89)		1327(42.34)	1230(42.55)	
Drinking			< 0.001			< 0.001
Current	196(4.67)	239(7.75)		662(21.12)	904(31.27)	
Ever	205(4.88)	144(4.67)		738(23.55)	469(16.22)	
Never	3797(90.45)	2702(87.59)		1734(55.33)	1518(52.51)	
Exercise			< 0.001			< 0.001
No	3272(77.94)	2035(65.96)		2142(68.35)	1590(55.00)	
Yes	926(22.06)	1050(34.04)		992(31.65)	1301(45.00)	
Social activity			< 0.001			< 0.001
No	1702(40.54)	898(29.11)		951(30.34)	571(19.75)	
Yes	2496(59.46)	2187(70.89)		2183(69.66)	2320(80.25)	
Education (years)			0.001			< 0.001
0	2493(59.39)	1722(55.82)		777(24.79)	566(19.58)	
1–6	797(18.99)	697(22.59)		1154(36.82)	1102(38.12)	
≥ 7	908(21.63)	666(21.59)		1203(38.39)	1223(42.30)	

### Multivariate analysis of SRH and gender differences

Table 3 shows the Logistic regression analysis results on the SRH reported by Chinese older male and female adults. Residence, BMI, self-reported income, drinking, exercise, and social activity were the factors influencing SRH as reported by male and female respondents. Meanwhile, gender differences existed in SRH-influencing factors between older female and male adults, with ’70–79 years old’, ’80–89 years old’, ‘widowed’, ‘never smoking’, and ‘≥7 years of education’ reaching the level of significance only in older female adults and ‘rural’ and ‘ever smoking’ reaching the level of significance only in older male adults.

**Table 3:** Logistic regression analysis of SRH reported by male and female respondents.

Variable	Female			Male		
	*P*	*OR*	[95%*CI*]	*P*	*OR*	[95%*CI*]
Residence						
City		1.000			1.000	
Town	0.006	0.822	(0.714,0.946)	0.013	1.212	(1.041,1.410)
Rural	0.408	0.945	(0.827,1.080)	0.001	1.290	(1.114,1.495)
Age (years)						
< 70		1.000			1.000	
70–79	0.011	0.787	(0.654,0.948)	0.390	0.923	(0.768,1.108)
80–89	0.008	0.763	(0.626,0.931)	0.176	0.876	(0.723,1.061)
90–99	0.285	0.887	(0.713,1.105)	0.401	0.912	(0.736,1.131)
≥ 100	0.387	0.903	(0.715,1.139)	0.693	0.947	(0.723,1.241)
Marital status						
Married and living with spouse		1.000			1.000	
Widowed	0.006	1.217	(1.059,1.398)	0.248	1.087	(0.943,1.253)
Other	0.738	0.932	(0.618,1.407)	0.202	0.835	(0.632,1.102)
Living status						
Living with family		1.000			1.000	
Alone	0.197	0.915	(0.800,1.047)	0.540	1.056	(0.887,1.257)
Nursing home	0.247	0.845	(0.635,1.124)	0.883	1.026	(0.728,1.446)
BMI (kg/m^2^)						
18.5–23.9		1.000			1.000	
< 18.5	0.004	0.820	(0.717,0.938)	0.002	0.765	(0.646,0.906)
24.0-27.9	0.802	1.016	(0.895,1.154)	0.371	1.061	(0.932,1.208)
≥ 28.0	0.807	1.022	(0.860,1.215)	0.555	1.062	(0.870,1.297)
Self-reported income						
Poor		1.000				
So so	< 0.001	2.289	(1.910,2.744)	< 0.001	2.646	(2.162,3.237)
Rich	< 0.001	4.715	(3.825,5.812)	< 0.001	5.563	(4.432,6.983)
Smoking						
Current		1.000			1.000	
Ever	0.197	0.800	(0.570,1.122)	0.007	0.819	(0.708,0.947)
Never	0.019	0.749	(0.588,0.954)	0.446	0.948	(0.827,1.087)
Drinking						
Current		1.000			1.000	
Ever	< 0.001	0.567	(0.421,0.763)	< 0.001	0.495	(0.420,0.583)
Never	< 0.001	0.585	(0.477,0.719)	< 0.001	0.699	(0.612,0.799)
Exercise						
No		1.000			1.000	
Yes	< 0.001	1.535	(1.366,1.724)	< 0.001	1.517	(1.350,1.704)
Social activity						
No		1.000			1.000	
Yes	< 0.001	1.514	(1.347,1.702)	< 0.001	1.451	(1.270,1.659)
Education (years)						
0		1.000			1.000	
1–6	0.488	1.048	(0.918,1.197)	0.101	1.131	(0.977,1.309)
≥ 7	0.005	0.826	(0.724,0.943)	0.432	1.063	(0.913,1.238)

### FDA results

We performed a quantitative analysis of the contribution level of different influencing factors to explain the differences in SRH reported by Chinese older male and female adults. The specific results for the decomposition of SRH differences are shown in Table 4. The results of the FDA showed that 86.65% of the differences in SRH were caused by the observed variables, whereas 13.35% of the differences were caused by gender differences and unmeasured variables. Among the variables causing the explained part of the differences, self-reported income, smoking, drinking, exercise, social activity, and education were influencing factors that reached the level of significance (p < 0.05), with contribution levels of 15.28%, 32.70%, 42.49%, 17.41%, 15.10%, and − 14.59% respectively.

**Table 4:** FDA of SRH reported by different genders.

Terms of decomposition	SRH			
Difference	-0.06491378			
Explained (%)	-0.05624484(86.65%)			
Non-explained (%)	-0.00866894(13.35%)			
Explained				
Contribution to difference	*P*	$\beta$	Contribution (%)	[95%*CI*]
Residence	0.747	-0.0001196	0.18	(-0.0008458,0.0006066)
Age	0.955	0.0001094	-0.17	(-0.0037019,0.0039207)
Married status	0.071	0.0065243	-10.05	(-0.0005621,0.0136106)
Living status	0.111	-0.0010795	1.66	(-0.0024079,0.0002489)
BMI	0.856	0.0000215	-0.03	(-0.0002114,0.0002544)
Self-reported income	< 0.001	-0.0099165	15.28	(-0.01123,-0.008603)
Smoking	0.032	-0.0212254	32.70	(-0.040646,-0.0018048)
Drinking	< 0.001	-0.0275815	42.49	(-0.0396938,-0.0154692)
Exercise	< 0.001	-0.0113021	17.41	(-0.0142336,-0.0083705)
Social activity	< 0.001	-0.0098027	15.10	(-0.0125949,-0.0070104)
Education	0.021	0.0094731	-14.59	(0.0014138,0.0175324)

## Discussion

This study examined the factors influencing SRH reported by Chinese older male and female adults and performed a quantitative analysis of the contribution level of various influencing factors to explain these gender differences. The FDA is highly interpretable and can offer theoretical references to help create policies for older adults’ health concerns by relevant departments.

The study results suggested notable gender differences in SRH among Chinese older adults, with worse SRH reported by women than men. However, older female adults have a longer life expectancy than men. Our study results align with the findings of other researchers [41–44], namely the ‘gender paradox’ prevalent in health assessments worldwide. The differences were found by many other studies [3, 45, 46] and existed in Colombia [47], Abu Dhabi [48], India [5] and so on. But the researchers in America [8, 9] didn’t observe it.

Regarding gender differences analysis, there existed gender differences in SRH-influencing factors between older female and male adults, with ’70–79 years old’, ’80–89 years old’, ‘widowed’, ‘never smoking’, and ‘≥7 years of education’ reaching the significance level only in women and ‘rural’ and ‘ever smoking’ only in men. Notably, the ‘marital status’ and ‘education’ variable reached the significance level only in women. The ‘widowed’ variable was the protected variable of SRH reported by older female adults, while ‘≥7 years of education’ was the risk variable, which were not consistent with the findings of other researchers [42, 46, 49]. This result suggested that the impact of marital status on SRH in Chinese older adults differed from that in other countries’ populations. The results on other variables were relatively consistent with the findings of other researchers [50–52]. For example, increased body weight, increased income, regular exercise, and social activities contributed to better SRH.

The results of the FDA suggested that self-reported income (15.28%), smoking (32.70%), drinking (42.49%), exercise (17.41%), social activity (15.10%), and education (-14.59%) were the variables influencing gender differences in SRH and reached the significance level. This finding suggested that drinking (42.49%), smoking (32.70%), and exercise (17.41%) were the most significant factors causing gender differences in SRH. Given that drinking, smoking and exercise reflected an individual’s daily lifestyle habits, we could conclude that varied lifestyle habits led to gender differences in health status. This was a crucial finding and served as a reference for relevant departments to eradicate gender differences in health among Chinese older adults from a more holistic perspective.

Moreover, our study results have substantial policy implications. First, the government should focus on health issues of older female adults of advanced age, especially older adults with poor economic conditions and low social status. Second, the government should promote education on smoking and drinking policies to encourage the adoption of a healthy lifestyle among older adults. Third, required fitness facilities and guidance must be provided in communities to help older adults develop an awareness of regular exercise. The gender differences in health between Chinese older male and female adults are not solely caused by lifestyle choices. Attention must be paid to various other factors, including education and social support.

Our study also has a few limitations. First, our study used data from CLHLS in 2018, a cross-sectional study not inclusive of older adults in all regions of China. Second, SRH is a subjective measure of health and cannot objectively reflect the health status of the respondents. Lastly, the SRH of older adults is influenced by several factors, and we have only measured some of these. Although our study has these limitations, our results may offer new insights into the gender health differences among older Chinese adults. In subsequent studies, we will collect more data and include more factors in our analysis to verify the validity of our results.

## Conclusions

This study examined the gender differences in SRH, with older male adults reporting significantly better SRH than older female adults. The most significant reasons for gender differences included drinking, smoking, exercise, self-reported income, social activity, and education, and the Chinese government must focus on these factors to reduce the gender health gap. Our study results can help implement the Healthy China initiative, inform intervention measures, and offer new proposals on the making of policies on older adults’ health issues by the Chinese government to improve health equity.

### Electronic supplementary material

Below is the link to the electronic supplementary material.

Supplementary Material 1

## Data Availability

The datasets analysed during the current study are available in the Peking University Open Research Data repository, https://opendata.pku.edu.cn/file.xhtml?fileId=10356&version=2.1.

## List of Abbreviations

CLHLS: Chinese longitudinal healthy longevity survey
SRH: Self-rated health
BMI: Body mass index
ADL: Activities of daily living
SES: Socioeconomic status
FDA: Fairlie decomposition analysis
OR: Odds ratios
CI: Confidence interval
