# Supplementary material for: Decomposition and comparative analysis of health inequities between the male and female older adults in China: a national cross-sectional study
Source: BMC Public Health. 2023 Oct 19;23:2045. doi: 10.1186/s12889-023-15814-5 (PMC10588259; doi:10.1186/s12889-023-15814-5)
Supplement: Supplementary file 1 — Supplementary Material 1 [file 12889_2023_15814_MOESM1_ESM.doc]

Table A1 Variable definitions and values assigned to different variables.

| Type | Name | Assignment |
| --- | --- | --- |
| Dependent variable | SRH | Bad SRH=0, Good SRH=1 |
| Grouping variable | Gender | Female=0, Male=1 |
| Demographic and sociological characteristics | Age (years) | <70=0, 70-79=1, 80-89=2, 90-99=3, ≥100=4 |
|  | Marital status | Married and living with spouse=0, Widowed=1, Other^*^=2 |
|  | Living status | Living with family=0, Alone=1, Nursing home=2 |
|  | BMI (kg/m^2^) | <18.5=0, 18.5-23.9=1, 24.0-27.9=2, ≥28.0=3 |
|  | Smoking | Current=0, Ever=1, Never=2 |
|  | Drinking | Current=0, Ever=1, Never=2 |
|  | Exercise | No=0, Yes=1 |
|  | Social activity | No=0, Yes=1 |
| Socioeconomic status | Self-reported income | Poor=0, So so=1, Rich=2 |
|  | Residence | City =0, Town=1, Rural =2 |
|  | Education (years) | 0=0, 0-6=1, ≥7=2 |

^*^other: include married but not living with spouse, divorced and never married.

Table A2 Logistic regression analysis of SRH reported by male and female respondents (95 individuals were excluded due to age<65 years old. 142 individuals were excluded due to non-response to SRH indicators. 58 individuals were excluded because of more than 20% missing values. And we did multiple interpolation of the missing values of the independent variables. Finally, this study only excluded 296(1.86%) individuals and included 15578 individuals).

| Variable | Female | | | Male | | |
| --- | --- | --- | --- | --- | --- | --- |
|  | *P* | *OR* | [95%*CI*] | *P* | *OR* | [95%*CI*] |
| Residence |  |  |  |  |  |  |
| City |  | 1.000 |  |  | 1.000 |  |
| Town | 0.033 | 0.869 | (0.764,0.989) | 0.021 | 1.183 | (1.026,1.365) |
| Rural | 0.811 | 1.015 | (0.898,1.148) | 0.001 | 1.276 | (1.110,1.466) |
| Age |  |  |  |  |  |  |
| <70 |  | 1.000 |  |  | 1.000 |  |
| 70-79 | 0.012 | 0.797 | (0.667,0.952) | 0.532 | 0.945 | (0.792,1.128) |
| 80-89 | 0.007 | 0.772 | (0.639,0.932) | 0.188 | 0.884 | (0.735,1.062) |
| 90-99 | 0.087 | 0.834 | (0.678,1.027) | 0.450 | 0.924 | (0.753,1.134) |
| >100 | 0.172 | 0.859 | (0.691,1.068) | 0.554 | 0.926 | (0.719,1.193) |
| Marital status |  |  |  |  |  |  |
| Married and living with spouse |  | 1.000 |  |  | 1.000 |  |
| Widowed | 0.012 | 1.183 | (1.038,1.348) | 0.510 | 1.047 | (0.913,1.200) |
| Other | 0.440 | 0.861 | (0.588,1.260) | 0.195 | 0.841 | (0.648,1.093) |
| Living status |  |  |  |  |  |  |
| Living with family |  | 1.000 |  |  | 1.000 | (0.913,1.282) |
| Alone | 0.527 | 0.960 | (0.846,1.089) | 0.363 | 1.082 | (0.699,1.275) |
| Nursing home | 0.040 | 0.774 | (0.605,0.988) | 0.706 | 0.944 |  |
| BMI (kg/m^2^) |  |  |  |  |  |  |
| 18.5-23.9 |  | 1.000 |  |  | 1.000 |  |
| <18.5 | 0.012 | 0.854 | (0.755,0.966) | 0.005 | 0.791 | (0.673,0.930) |
| 24.0-27.9 | 0.900 | 0.993 | (0.882,1.116) | 0.133 | 1.099 | (0.971,1.244) |
| ≥28.0 | 0.765 | 0.975 | (0.824,1.153) | 0.909 | 1.011 | (0.837,1.222) |
| Self-reported income |  |  |  |  |  |  |
| Poor |  | 1.000 |  |  |  |  |
| So so | <0.001 | 2.423 | (2.048,2.867) | <0.001 | 2.795 | (2.305,3.390) |
| Rich | <0.001 | 4.944 | (4.075,5.997) | <0.001 | 5.700 | (4.589,7.080) |
| Smoking |  |  |  |  |  |  |
| Current |  | 1.000 |  |  | 1.000 |  |
| Ever | 0.093 | 0.763 | (0.556,1.046) | 0.001 | 0.790 | (0.687,0.907) |
| Never | 0.004 | 0.716 | (0.570,0.899) | 0.147 | 0.908 | (0.796,1.035) |
| Drinking |  |  |  |  |  |  |
| Current |  | 1.000 |  |  | 1.000 |  |
| Ever | <0.001 | 0.544 | (0.411,0.720) | <0.001 | 0.498 | (0.426,0.583) |
| Never | <0.001 | 0.599 | (0.494,0.726) | <0.001 | 0.694 | (0.610,0.789) |
| Exercise |  |  |  |  |  |  |
| No |  | 1.000 |  |  | 1.000 |  |
| Yes | <0.001 | 1.605 | (1.438,1.792) | <0.001 | 1.528 | (1.367,1.709) |
| Social activity |  |  |  |  |  |  |
| No |  | 1.000 |  |  | 1.000 |  |
| Yes | <0.001 | 1.674 | (1.502,1.866) | <0.001 | 1.559 | (1.374,1.769) |
| Education (years) |  |  |  |  |  |  |
| 0 |  | 1.000 |  |  | 1.000 |  |
| 1-6 | 0.291 | 1.069 | (0.944,1.211) | 0.200 | 1.094 | (0.953,1.257) |
| >=7 | 0.002 | 0.827 | (0.733,0.934) | 0.441 | 1.058 | (0.917,1.221) |

Table A3 FDA of SRH reported by different genders (after multiple interpolation).

| Terms of decomposition | SRH | | |
| --- | --- | --- | --- |
| Explained | | | |
| Contribution to difference | *P* | *β* | [95%*CI*] |
| Residence | 0.376 | 0.0002746 | (-0.0003337,0.0008828) |
| Age | 0.573 | -0.0011516 | (-0.0051567,0.0028536) |
| Married status | 0.137 | 0.0051136 | (-0.0016202,0.0118474) |
| Living status | 0.082 | -0.001036 | (-0.0022036,0.0001316) |
| BMI | 0.790 | -0.0000124 | (-0.0001035,0.0000788) |
| Self-reported income | <0.001 | -0.0097705 | (-0.0110232,-0.0085178) |
| Smoking | 0.011 | -0.0229912 | (-0.0407502,-0.0052321) |
| Drinking | <0.001 | -0.0236659 | (-0.0346451,-0.0126867) |
| Exercise | <0.001 | -0.0132202 | (-0.0161739,-0.0102666) |
| Social activity | <0.001 | -0.0150722 | (-0.0182071,-0.0119373) |
| Education | 0.011 | 0.0096336 | (0.0021927,0.0170744) |
